# Hungry for more: Australian medical students’ competence, attitudes and preferences towards nutrition education

**DOI:** 10.1186/s12909-022-03748-2

**Published:** 2022-09-27

**Authors:** Jacqueline Bredhauer, Sam Cone, Lucy Brown, Genevieve Moseley, Alyce Wilson, Robyn Perlstein, Lauren Ball

**Affiliations:** 1 School of Medicine, Monash University, Melbourne, 3800 Australia; 2 JMO Unit, Hunter New England Local Health District, NSW Health, Newcastle, 2300 Australia; 3 School of Medicine, The University of Adelaide, Adelaide, 5005 Australia; 4 IMPACT Strategic Research Centre, School of Medicine, Deakin University, Geelong, 3220 Australia; 5 Maternal, Child and Adolescent Health Program, Burnet Institute, Melbourne, 3004 Australia; 6 Nossal Institute of Global Health, School of Population and Global Health, University of Melbourne, Melbourne, 3010 Australia; 7 School of Medicine, Deakin University, Geelong, 3220 Australia; 8 Menzies Health Institute Queensland, Griffith University, Southport, 4222 Australia

**Keywords:** Nutrition, NCDs, Culinary medicine, Medical education, Public health

## Abstract

**Background:**

Inadequate nutrition education in medical training is a prevailing global challenge. This study assessed Australian medical students’ self-perceived competencies in nutrition and preferences regarding nutrition education in medical training.

**Methods:**

We conducted a national cross-sectional online survey between September 2019 and January 2020. Our survey collected sociodemographic characteristics and assessed nutrition competency according to a validated assessment tool. All Australian medical students aged over 18 were eligible to participate.

**Results:**

One hundred ninety-five medical students representing 20 Australian medical schools completed the survey and reported moderate nutrition knowledge (17·6 ± 4.1 out of 35, 50%) and skills (29.8 ± 7.6 out of 55, 54%). Students demonstrated positive attitudes towards nutrition training/education (35·9 ± 4.0 out of 40, 90%). Most medical students (*n* = 148, 72%) reported they had sought some form of nutrition education outside of their degree. Students showed preference for practical, evidence-based nutrition education that is integrated in and prioritised throughout medical training.

**Conclusions:**

Australian medical students express positive attitudes towards nutrition but report only low to moderate nutrition knowledge and skills. There is an opportunity to incorporate practical, regular nutrition learning activities into Australian medical curriculums to equip future doctors to adequately address non-communicable disease. Such initiatives are likely to be well received by students.

## Background

There is an urgent need to improve global diets and food systems in order to safeguard human and planetary health [1, 2]. In 2017, poor nutrition was associated with 11 million deaths and the loss of 255 million disability-adjusted life years, making it the leading modifiable risk factor for morbidity and mortality globally [1]. Unhealthy diets are a key driver of the global non-communicable disease (NCD) epidemic [1, 3]. In association with an increasing prevalence of high energy density and poor quality diets, 2.1 billion adults world-wide are experiencing overweight or obesity [2, 4]. Furthermore, 821 million people remain undernourished, and two billion have micronutrient deficiencies due to low diet quality [5]. Addressing risk factors such as diets high in sodium, low in wholegrains, and low in fruits can significantly improve the health of individuals [1–3]. Nutrition must be a central component of national NCD preventative strategies and universal health coverage roadmaps [6]. The environmental implications of food systems mean that poor nutrition also places an increasing burden on planetary health [2]. A sustainable approach to nutrition is therefore required [2].

Primary care doctors are often the first point of contact patients have with health-care systems [7]. These doctors are thus ideally placed to help patients improve their diets [8]: they can assess dietary risk, provide support and education, and arrange follow-up and appropriate referrals [7]. However, doctors report rarely discussing nutrition even in consultations with patients who are overweight [9], with only 3–4% of general practitioner consults in Australia involving discussion about nutrition [10]. Barriers to discussing nutrition include insufficient time, unclear understanding of their role and low self-efficacy in nutrition [7, 8]. Measures to improve doctors’ nutrition competence are warranted. However, there is a well-established, widespread deficiency of nutrition education in medical curriculums [11]. There are increasing calls for clinically relevant nutrition education for medical students in order to address the global burden of non-communicable disease [8]. Medical student perspectives are important in guiding the implementation of nutrition education into crowded medical school curriculums, and medical students value opportunities to be involved in curriculum change [12]. This study aims to assess the self-perceived nutrition competence of Australian medical students, and to identify their priorities regarding nutrition education.

## Methods

We conducted a cross-sectional online survey of Australian medical students. Our study received ethical approval from Griffith University Human Research Ethics Committee (GU Ref No: 691, 2019).

### Participants and recruitment

All students enrolled in an Australian undergraduate or postgraduate medicine course and aged greater than 18 years were eligible to participate in this study. All medicine courses in Australia are accredited by the Australian Medical Council according to strict standards and a specific set of graduate outcomes [13], hence students have comparable experiences across different universities and at either the undergraduate or postgraduate level.

Participants were recruited by convenience and snowball sampling, and provided their consent for inclusion via an online form at the commencement of the survey. We recruited most students through the Australian Medical Students’ Association (AMSA). AMSA is the peak representative body for Australia’s 17,000 medical students from twenty-two medical schools and advocates for medical students’ rights, improved curriculum and more broadly for improved public health outcomes in Australia and globally. AMSA promoted the online survey via social media (Facebook and Twitter), email, and through individual university representatives. Nutrition in medicine special interest groups also shared the survey link through Facebook and Instagram pages.

We offered the chance to win one of five AUD30 shopping gift vouchers through an online prize draw. Participants wishing to enter the draw were given the option of providing their email address at the end of the survey.

### Survey instrument

The online survey comprised the validated nutrition competence tool (NUTCOMP) [14], assessing individuals’: (a) self-perceived confidence in knowledge about nutrition and chronic disease; (b) confidence in nutrition skills; (c) confidence in communication and counselling about nutrition; (d) attitudes towards nutrition care and; (e) preferences regarding potential approaches to nutrition education in medical school [14] (Table 1). The first four survey sections used a 5-point Likert scale to rate confidence in all items relevant for each question [14]. We also collected information regarding external nutrition training, and invited participants to provide suggestions for nutrition education approaches via an open-ended question. Finally, we collected sociodemographic characteristics including, year level and medical school.

**Table 1:** Sample NUTCOMP survey questions [14]

NUTCOMP Domain	Sample question
Knowledge	“Please rate how confident you are in your knowledge of how different body systems are affected by food and nutrients”
Skills	“Please rate how confident you are in your ability to use the Australian Guide to Healthy Eating to evaluate the appropriateness of an individual’s food intake”
Communication and counselling	“Please rate how confident you are in your ability to clearly describe what patients/clients can expect from their discussions with you about food or nutrition”
Attitudes	“Please rate your agreement with the following statement: providing specific nutrition recommendations to my patients/clients that can assist with managing their chronic disease is an effective use of my professional time”

Before commencing data collection, we conducted a pilot survey with five AMSA members who provided feedback on question wording and interpretation. The final survey took approximately 15–20 minutes to complete. Survey data was collected between September 2019 and January 2020.

### Data analysis

We analysed quantitative data using Qualtrics survey software™ and SPSS statistics software™ (IBM 2009). For each of the NUTCOMP sections, scores were calculated by summing the value assigned to each answer (not at all confident = 1; not very confident = 2; somewhat confident = 3; very confident = 4, and extremely confident = 5). Attitude scores were calculated (completely disagree = 1; somewhat disagree = 2; neither agree nor disagree = 3; somewhat agree = 4, and completely agree = 5). We compared the responses of undergraduate and postgraduate participants using Pearson’s Chi-Squared tests. Finally, we conducted thematic analysis [15] of qualitative data using NVivo software™ (QSR 2020).

## Results

The survey was completed by 195 medical students, although 358 commenced the survey. Most of the respondents were female (*n* = 151, 77%). There was a broad representation of training levels, with just under half (*n* = 89, 46%) of students in Pre-Clinical training, just under half (*n* = 89, 46%) in Clinical training and the remaining (*n* = 17, 9%) in mixed training programs (combination of Pre-Clinical and Clinical). Twenty of the 22 Australian medical schools were represented. Table 2 outlines the demographic characteristics of respondents.

**Table 2:** Demographic characteristics of participants (*n* = 195 responses)

Demographics	N (%)
**Gender**	
Male	40 (21%)
Female	151 (77%)
Unspecified	4 (2%)
**Years of age**	
17–19 years	15 (8%)
20–22 years	75 (38%)
23–25 years	67 (34%)
26–30 years	29 (15%)
31–40 years	9 (5%)
**Type of medical degree**	
Undergraduate	90 (46%)
Postgraduate	105 (54%)
**Medical school**	
Monash University	36 (18%)
Griffith University	35 (18%)
Deakin University	19 (10%)
University of Melbourne	14 (7%)
University of New South Wales	12 (6%)
University of Queensland	12 (6%)
University of New England	11 (6%)
The University of Adelaide	9 (5%)
University of Newcastle	7 (4%)
The University of Western Australia	7 (4%)
Sydney University	6 (3%)
James Cook University	5 (3%)
Western Sydney University	4 (2%)
University of Tasmania	4 (2%)
University of Notre Dame, WA	4 (2%)
University of Wollongong	3 (2%)
Bond University	3 (2%)
Flinders University	2 (1%)
Australian National University	1 (1%)
Curtin University	1 (1%)
University of Notre Dame, NSW	0 (0%)
Macquarie University	0 (0%)
**Year of Medicine**	
1st	30 (15%)
2nd	68 (35%)
3rd	29 (15%)
4th	46 (24%)
5th	19 (10%)
6th	3 (2%)
**Type of Medical Training**	
Pre-Clinical	89 (46%)
Clinical	89 (46%)
Mixed	17 (9%)
Total	195

Table 3 describes participants’ nutrition competence scores. Self-reported knowledge of nutrition was moderate, with a mean score of 17·6 ± 4.1 out of 35 (50%). Most respondents regarded themselves as not very confident (2/5) in their nutritional skills, with a mean score of 29.8 ± 7.6 out of 55 (54%). Positive attitudes towards the importance of nutrition were clear, with a mean score of 35·9 ± 4.0 out of 40 (90%). Over two thirds of students (*n* = 148, 72%) reported they had previously sought some form of additional nutrition education, such as self-directed reading or training outside of their medical degree. There were no statistically significant differences in the responses of undergraduate medicine students compared to postgraduate medicine students across all NUTCOMP domains.

**Table 3:** Nutrition competence scores of Australian medical students (*n* = 195)

Nutrition Competence Construct	Type of Medical Degree	n	Mean (SD)	*P*-value
**Knowledge** **(Maximum = 35)**	Undergraduate	90	17.7 (4.0)	0.90
	Post Graduate	105	17.6 (4.2)	
	All students	195	17.6 (4.1)	
**Skills** **(Maximum = 55)**	Undergraduate	90	29.8 (7.0)	0.99
	Post Graduate	105	29.8 (8.2)	
	All students	195	29.8 (7.6)	
**Communication** **(Maximum = 45)**	Undergraduate	90	29.0 (6.4)	0.47
	Post Graduate	105	28.3 (6.7)	
	All students	195	28.7 (6.5)	
**Attitudes** **(Maximum = 40)**	Undergraduate	90	35.9 (3.9)	0.94
	Postgraduate	105	36.0 (4.1)	
	All students	195	35.9 (4.0)	

A small group of respondents (*n* = 48, 23%) provided suggestions about how the content and delivery of nutrition education could be improved for future medical students. Qualitative analysis revealed the following themes: content integration, hands-on learning, evidence-based practice, and social context. Students acknowledged the importance of evidence-based and clinically relevant nutrition education, with the need for content to be integrated throughout medical degrees into existing pre-clinical and clinical learning.

Four students emphasised the importance of practical, hands on learning, such as incorporating nutritional counselling into bedside tutorials, or learning through cooking ‘culinary medicine’. One undergraduate student explained: “...cooking class [es]... would be both a visual way to learn, but also important for the students’ health” (F, 23–25 years). Students also expressed a desire to develop skills in nutrition counselling and communication. One second-year post-graduate student explained: *“We need to learn how to non-judgmentally enquire about [a] patient’s diet and [how to] motivational [ly] interview for them to change to healthier habits”* (F, 20–22 years).

Students believed nutrition teaching should be focused on chronic disease prevention and management, incorporating specific and individualised diets where appropriate. A fifth-year undergraduate student suggested: *“More focus on actual evidence-based advice to give to patients rather than just the building blocks of nutrition”* (F, 20–22 years). Respondents also felt it would be important to gain an understanding of professional roles and scope of nutrition practice, suggesting “a range of presenters with different backgrounds” (M, 31–40 years). Students considered skills in critical appraisal of nutrition evidence – including understanding the role of specific diets, identifying and managing industry bias, and tackling misinformation – to be important. One undergraduate student emphasised the importance of “*nutrition in the age of social media and misinformation*” (F, 18–19 years).

A number of participants expressed a strong interest in a body-positive, “health at every size” (F, 20–22 years) approach to nutrition, and felt that the recognition and management of eating disorders should be included in course content. Finally, students expressed an interest in understanding nutrition from a public health lens, indicating an interest in topics such as advocacy and food sustainability.

## Discussion

This study employed a cross-sectional design to understand competencies and attitudes regarding nutrition and nutrition education among Australian medical students. Undergraduate and postgraduate medicine students demonstrated low confidence in nutrition yet positive attitudes towards the role of nutrition in healthcare and a keen interest to learn more about the topic. Students want nutrition teaching to be practical, clinically relevant, evidence-based and socially conscious. These key findings support the inclusion and reform of nutrition education in Australian and global medical curriculums.

Our survey indicates that nutrition competency amongst Australian medical students is lacking. Survey respondents reported moderate knowledge of nutrition, and low confidence in nutrition skills. This is an expected outcome given that the Australian Medical Council does not currently include nutrition among its 90 curriculum attributes nor in its proposed assessment items for medical schools [16]. Without nutrition training, future Australian doctors are not adequately equipped to respond to chronic illness, including this country’s leading cause of disease burden – coronary heart disease [17]. Our findings reflect global trends: research spanning 50 years has revealed a long-standing deficiency of high-quality nutrition education in medical curriculums globally, undermining the medical profession’s ability to address the growing burden of non-communicable disease [11]. The recent recognition of nutritional care - including preventative nutrition - as a human right, emphasises the importance of equipping future doctors with nutritional skills [18].

Our survey also suggests that Australian medical students recognise the importance of nutrition in healthcare. Participants revealed positive attitudes towards nutrition. Most respondents reported seeking nutrition education and training outside of their medical degree, demonstrating high levels of motivation to obtain nutrition training among this group. Comparable findings were documented in a recent survey of UK medical students’ and doctors’ views surrounding nutrition in medical education, which revealed a majority consensus on the importance of nutrition in health (> 90%) and of the provision of nutrition care by medical professionals (> 95%) [19]. In line with study, many UK medical students indicated a desire for more nutrition education [19]. Medical students want high-quality nutrition education. Curriculum change would likely be well-received.

Students indicated a preference for nutrition teaching to be practical and clinically relevant. Those who provided curriculum suggestions placed importance on nutrition teaching that is clinically focused, incorporates counselling and communication skills, and includes practical components such as learning through cooking. Indeed, a review of nutrition curriculum initiatives in the US, Crete, the UK and Israel recommended combined clinical and practical nutrition courses [8]. Pilots of ‘culinary medicine’ courses, a form of teaching that blends cooking with nutrition science and medicine, have been well-received and effective in the United States [20, 21]. Further exploration of ‘culinary medicine’ as a practical way to start incorporating nutrition education into medical curricula is warranted.

A global deficiency in nutrition training in medical education has been well recognised [11]. Our findings emphasise the skills and knowledge gap in nutrition amongst a broad sample of Australian medical students from twenty of Australia’s twenty-two medical schools. We also present the current priorities of a subset of students in relation to the nature of the nutrition training they receive. We recognise that we are limited by selection bias: medical students who are more interested in nutrition may have been more likely to undertake our survey. Further, the 48 participants who volunteered curriculum suggestions were likely a particularly motivated group. Our population generalisability is limited by a small sample size and a homogenous sample. Most respondents were female (78%) in comparison with approximately 52% in the general medical student population [22]. However, our study provides a unique starting point for nutrition education implementation from the perspective of current medical students.

This small-scale survey highlights gaps in nutrition teaching in Australian medical schools, and medical student interest in accessing clinically relevant nutrition teaching. Further research is required to explore these trends amongst larger groups of students, and to pilot nutrition teaching initiatives. We put forward three calls to action in light of our findings. Firstly, we call upon Medical Deans’ Associations to make nutrition education compulsory in medical training so as to equip medical professionals to adequately respond to the global burden of NCDs. We recommend that medical curricula in Australia and globally adopt a Nutrition Competency Framework spanning the prevention and treatment of diseases relating to nutrition, in line with benchmarks developed by recent international interdisciplinary consensus [23]. Finally, we call upon researchers and universities to develop, pilot and assess innovative nutrition initiatives in medical curriculums, such as ‘culinary medicine’ programs.

## Conclusions

Poor nutrition is a leading risk factor for morbidity and mortality globally [1], yet medical training does not include adequate education in nutrition [11]. This survey reveals only low to moderate nutrition competency amongst Australian medical students, despite positive attitudes towards the role of nutrition in healthcare and a strong motivation to obtain nutrition education and training. This suggests a need for reform. Nutrition education should be practical and clinically orientated; mandatory; and included in accreditation competencies. This will equip the future medical workforce with the knowledge and skills to adequately prevent and manage diet related NCDs.

## Data Availability

The data collected for this study are available from the corresponding author on reasonable request.

## Abbreviations

NCDs: Non-communicable disease
AMSA: Australian Medical Student’s Association
NUTCOMP: Nutrition Competency Tool
